# Long-Term Survival after Treatment with Induction Therapy and Surgery for Mediastinal Carcinoma of Unknown Primary

**DOI:** 10.70352/scrj.cr.24-0011

**Published:** 2025-02-22

**Authors:** Yasushi Sakamaki, Naoya Takada, Yuya Kogita, Nanami Hiraiwa

**Affiliations:** Department of Thoracic Surgery, Osaka Keisatsu Hospital, Osaka, Osaka, Japan

**Keywords:** carcinoma of unknown primary, mediastinum, mediastinal adenocarcinoma, metastatic lymph node carcinoma, induction therapy

## Abstract

**INTRODUCTION:**

Carcinoma of unknown primary (CUP) is rarely located in the mediastinum. Most cases are revealed to be metastatic lymph node carcinoma, which carries a poor prognosis. The optimal treatment for CUP confined to the mediastinum is yet to be established, and the long-term outcome of induction therapy in combination with surgery for mediastinal CUP is unclear.

**CASE PRESENTATION:**

A 46-year-old man with no history of malignancy was diagnosed with anterior mediastinal adenocarcinoma through biopsy. The patient underwent chemoradiation for the tumor, which was initially suspected as invasive T4 lung cancer. After a favorable response to presurgical therapy, the tumor was deemed more likely a mediastinal tumor, and it was completely resected simultaneously with the thymus, the partial left lung, and the partial left innominate vein. The tumor contained histologic features identifiable as a lymph node tissue and lacked any thymic tissue, which led to the final diagnosis as metastatic lymph node adenocarcinoma; however, its origin was unknown. No signs of recurrence were detected for 13 years after surgery.

**CONCLUSIONS:**

Our case suggests that even patients with mediastinal CUP deemed an advanced disease can achieve long-term survival after undergoing induction therapy and definitive surgery.

## Abbreviations

CUP
: carcinoma of unknown primary
IHC
: immunohistochemical staining
MCUP
: mediastinal lymph node carcinoma of unknown primary
CK
: cytokeratin
CD
: cluster of differentiation
Pax
: paired box
NCAM
: neural cell adhesion molecule
LWND
: lobectomy with lymph node dissection
NGS
: next-generation sequencing

## INTRODUCTION

Carcinoma of unknown primary (CUP) is not an uncommon malignancy; however, it is rarely located in the mediastinum.^1–4)^ Mediastinal CUP is often revealed to be metastatic carcinoma in the mediastinal lymph nodes with an unknown primary site, and the prognosis of patients with a localized tumor reportedly depends on the resectability of the tumor.^5)^ We herein report a case of mediastinal CUP in which the patient underwent induction therapy followed by definitive surgery and achieved long-term survival postoperatively.

## CASE PRESENTATION

**Fig. 1:**
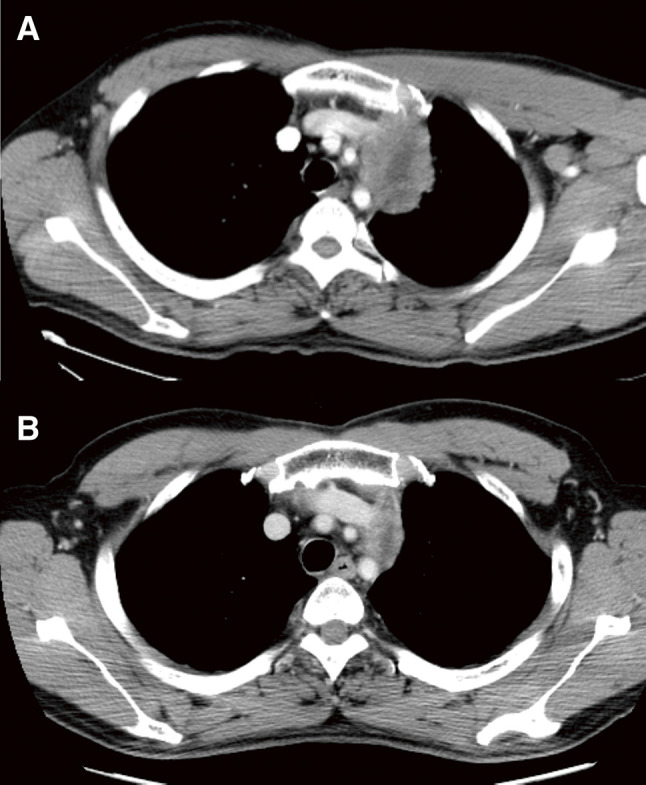
Computed tomography showed an anterior mediastinal tumor. The size was (**A**) 7.0 × 5.5 cm^2^ after diagnosis and (**B**) 3.8 × 2.0 cm^2^ after chemoradiotherapy. Tumor invasion to the left lung and left innominate vein was suspected. LIV, left innominate vein

**Fig. 2:**
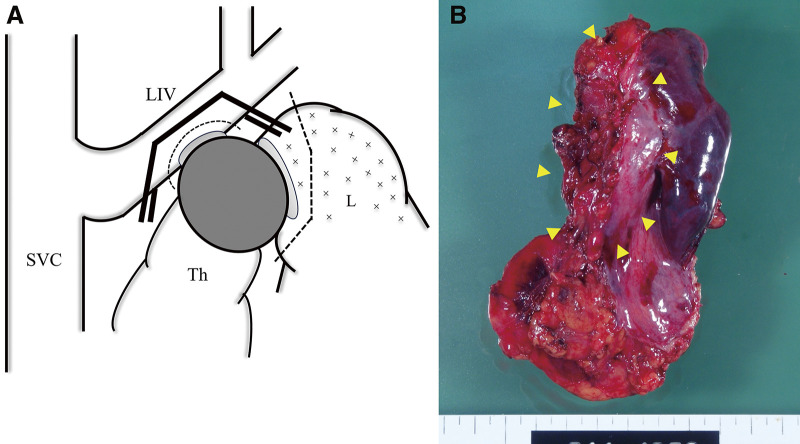
Schema of the surgical findings and gross appearance of the resected specimen. (**A**) SVC, superior vena cava; LIV, left innominate vein; Th, thymus; L, left lung. The tumor (dark gray) appeared to infiltrate (light gray) into the LIV and L. Dashed lines mean excision lines. (**B**) The tumor (arrowheads) was severely adherent to the left lung and left innominate vein (top, cranial; bottom, caudal).

**Fig. 3:**
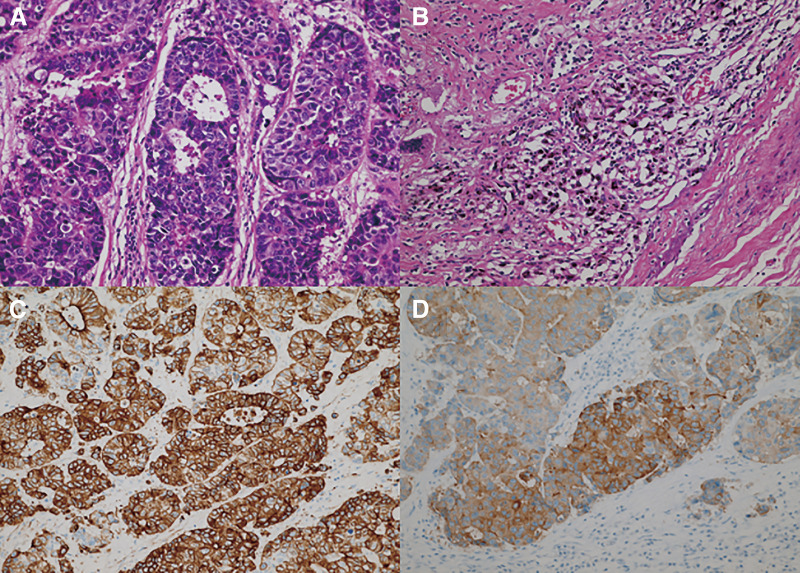
Histologic findings and immunoreactivity of the tumor cells (surgical specimen). Hematoxylin–eosin staining revealed (**A**) glandular luminal formations of atypical cells with (**B**) lymphocytic accumulation and surrounding carbon dust deposition. (**C**) Cytokeratin 7. (**D**) Synaptophysin.

A 46-year-old man with 90 pack-years of smoking and no history of malignancy was diagnosed with anterior mediastinal adenocarcinoma through biopsy. The tumor was deemed invasive and most likely T4 lung cancer originating in the left upper lobe on the basis of its appearance on computed tomography (**Fig. 1A**). No other lesions were detected in the metastatic workup using magnetic resonance imaging for the brain and positron emission tomography as a whole-body screening. Thymic carcinoma was included as a differential diagnosis. Although it was difficult to determine whether the tumor involved the mediastinal lymph nodes, the on-site treatment team comprising medical oncologists, radiologists, and thoracic surgeons approved a treatment plan for stage III non-small cell lung cancer. The patient underwent induction chemoradiotherapy (two courses of cisplatin plus vinorelbine with concurrent 40-Gy irradiation), which successfully debulked the tumor before definitive surgery (**Fig. 1B**). On the basis of the intraoperative final judgement that the tumor was more likely a mediastinal tumor than advanced lung cancer, we performed thymectomy with combined partial resection of the left lung and left innominate vein (LIV), which resulted in complete resection of the tumor. Through a trap-door thoracotomy, the tumor was uneventfully dissected from the left subclavian and common carotid arteries, with attention paid to the vagus nerve, after a wedge resection of the lung for suspected invasion. The site of the tumor that seemed to infiltrate into the LIV required en bloc excision with the venous wall using a partial occlusion clamp (**Fig. 2A**). The venous stump was sutured with a 5-0 polypropylene. The pathological examination on the resected specimen eliminated the lung as the primary site on the basis of its discontinuity with the tumor and confirmed that the simultaneously excised organs were severely adhered to the tumor but negative for tumor invasion (**Fig. 2B**). The tumor contained histologic features identifiable as lymph node tissue and lacked any thymic tissue, which led to the final diagnosis of adenocarcinoma in the mediastinal lymph node (**Figs. 3A**, **3B**). Immunohistochemical staining (IHC) did not reveal the origin of the tumor; therefore, the primary site remained unknown, and the tumor was diagnosed as CUP. The lung was included as a probable origin of the tumor cells, but the IHC results, which were incompatible with a typical lung cancer profile, could not definitely support the possibility. Briefly, positivity for cytokeratin (CK)7, synaptophysin (**Figs. 3C**, **3D**), and chromogranin A suggested pulmonary adenocarcinoma with neuroendocrine differentiation, while negativity for thyroid transcription factor 1 and napsin A suggested otherwise. Additionally, negativity for CD5 negated the possibility of thymic carcinoma. The tumor cells also showed negative reactions with CK20, CK14, p63, paired box (Pax)8, and neural cell adhesion molecule (NCAM). These findings were consistent between the surgically resected and pretreatment biopsy specimens. The patient has been well with no signs of recurrence detected for 13 years since surgery.

## DISCUSSION

CUP is defined as a metastatic tumor with an unknown primary site. It constitutes 3%–5% of all carcinomas and is among the 10 most frequent cancer diagnoses.^1)^ At its first appearance, CUP most commonly occurs in the lymph nodes, lungs, bone, and liver.^2–4)^ In cases of lymph node CUP, the first appearance predominantly occurs in the cervical nodes; occurrence in the mediastinal lymph nodes accounts for only 1.5% of all CUPs.^2,3)^ Of mediastinal lymph node CUP (MCUP), the most common histological type is adenocarcinoma (27%–40%), followed by squamous cell carcinoma (10%–17%) and neuroendocrine carcinoma.^2,5)^ Patients with MCUP are often characterized by the triad of male sex, smoker, and right-sided distribution,^5)^ of which our patient met two, excluding the affected side.

Patients with MCUP typically fall into one of the prognostically unfavorable subsets comprising 80% of all patients with CUP, as suggested by Pavlidis and Pentheroudakis.^1)^ This is presumably due to the common presentation of MCUP as one of multiple lesions, including those located in the pulmonary hilar lymph nodes, at the time of diagnosis.^4–6)^ However, the prognosis of patients with a localized tumor as in our case may be favorable if locoregional control is achieved by surgery or radiotherapy.^5,7)^ Fukushima *et al*.^5)^ reported much better survival for resected cases than unresectable cases (5-year overall survival rates of 75.1% and 28.6%, respectively) based on an analysis of 102 reported cases in Japan. Another recent study revealed better 5-year rates of intrathoracic control, freedom from distant metastasis, and overall survival in the occult primary site group (deemed MCUPs harboring a lung cancer profile) than those in the known primary site group in a cohort of patients treated with definitive radiotherapy for stage III non-small cell lung cancer.^7)^

Regarding multimodal treatments administered in combination with surgery for MCUP, adjuvant therapy is predominant, while induction therapy has rarely been reported; we found no patients with MCUP who received induction therapy in an online search of the English-language literature published during the last two decades.^4,8–14)^ Even if including CUPs in the mediastinum with no histological basis suggesting a metastatic lymph node, we found only two patients treated with induction therapy followed by definitive surgery.^15,16)^ Both of them had invasive large cell neuroendocrine carcinoma and underwent high-intensity induction therapy followed by technically challenging surgery, but survived for only 4–5 months postoperatively because of rapid progression of relapsing disease. A huge difference in the survival time between our patient and those in the reported cases^15,16)^ despite the same therapeutic regimen may be attributable to differences in the extension and histologic aggressiveness of carcinoma. The previous reports suggest that invasive neuroendocrine CUP is difficult to treat even with multidisciplinary therapy. Other relevant cases reported in the 21st century are summarized in **Table 1**, in which the lesion location is numbered according to the map published by the International Association for the Study of Lung Cancer.^17)^

**Table 1:** Reported cases of mediastinal lymph node carcinoma of unknown primary treated with multimodal therapy including surgery (2001–2023)

Case	Authors	Age (years)/sex	Histology	Location	Origin	Definitive surgery	Presurgical treatment	Adjuvant therapy	Outcome (months)
1	Riquet *et al*.^4)^	UI/M	La	4R + 10R	Unclear	Pnm + ND	None	RT	DOD (11)
2	Riquet *et al*.^4)^	UI/M	La	4R	Unclear	Lob + ND	None	CRT	NED (81)
3	Riquet *et al*.^4)^	UI/M	Ad	7 + 10L	Unclear	Pnm + ND	None	RT	NED (42)
4	Riquet *et al*.^4)^	UI/M	La	4R + 7 + 10R	Lung	Pnm+ND	None	RT	Died (7)
5	Riquet *et al*.^4)^	UI/M	Ad	7	Lung	Lob^#^ + ND	None	CT	NED (25)
6	Sorgho-Lougue *et al*.^8)^	41/M	Ad	5	NI	TR	None	RT	AWD (20)
7	Miwa *et al*.^9)^	72/M	Sq	1R + 2R	Unclear	TR + CVR + ND	None	RT	NED (82)
8	Miwa *et al*.^9)^	78/M	Ad	2R	NI	TR + ND	None	RT	NED (44)
9	Kobayashi *et al*.^10)^	55/M	UC	7	NI	TR	None	CT	NED (31)
10	Nakano *et al*.^11)^	68/M	Ad	3a^1^	Lung	TR + ND	CT^*^	None	NED (14)
11	Kawasaki *et al*.^12)^	40/M	Ad	6	Unclear	TR + Th	None	None	Recurred (64)
			Ad	5 + 7 + 10L^2^	Lung	Lob + ND	None	CT	AWD (59)
12	Nakano *et al*.^13)^	63/M	Ad	2R	Unclear	TR	None	CT	NED (37)
13	Shikata *et al*.^14)^	56/M	Ad	7	Lung	TR + ND	None	CRT	NED (36)
14	Present case	46/M	Ad	3a	Unclear	TR + CVR + Th	CRT^**^	None	NED (156)

Cases with hilar lesion alone were excluded. Patients’ ages in Cases 1 to 5 range from 49 to 73 years.^4)^ Location is numbered according to the International Association for the Study of Lung Cancer map.17) Origin refers to a putative primary site. Outcome contains postoperative survival time.

Ad, adenocarcinoma; AWD, alive with disease; CRT, chemoradiotherapy; CT, chemotherapy; CVR, combined vascular resection; DOD, died of disease; La, large cell carcinoma; Lob, lobectomy; M, male; ND, lymph node dissection; NED, no evidence of disease; NI, not identified; Pnm, pneumonectomy; RT, radiotherapy; Sq, squamous cell carcinoma; Th, thymectomy; TR, tumor resection; UC, undifferentiated carcinoma; UI, unidentifiable; 1R, right supraclavicular node; 2R, right upper paratracheal node; 3a, prevascular node; 4R, right lower paratracheal node; 5, subaortic node; 6, para-aortic node; 7, subcarinal node; 10L, left hilar node; 10R, right hilar node

^1^Relapsed after chemotherapy. ^2^Recurrent sites.

^*^Definitive therapy failed, ^**^Induction therapy, ^#^Performed for synchronous lymphoma.

The lung is occasionally considered the tumor origin of a CUP; pulmonary origin is reportedly identified in 20%–40% of MCUPs.^3–5)^ These exhibit a lung cancer profile, as shown by IHC, which is not totally compatible with the findings in our case. Furthermore, a recent study revealed that molecular profiling by next-generation sequencing (NGS), which can evaluate expression and alterations of selected genes, predicted the lung as the tumor origin in 21% of all analyzed CUPs.^18)^ Considering this information, whether tumor resection alone or surgery including lobectomy with lymph node dissection (LWND) is more appropriate for a resectable MCUP should be determined for each case on the basis of the tumor appearance and histological diagnosis, including IHC, if available. In reality, surgery with avoidance of LWND is currently the predominant technique unless a pulmonary lesion is identified.^9,11,13,14)^ Additionally, adjuvant therapy was reported to control the disease for some cases with a single-site MCUP in which the tumor was resected without LWND.^10,13,14)^ Notably, however, an unusual case of MCUP was reported in which the primary pulmonary lesion appeared with new metastasis to the ipsilateral mediastinal and hilar lymph nodes 5 years after resection of a single-site MCUP, eventually necessitating LWND.^12)^ In the future, novel analysis techniques, such as NGS, may predict the tumor origin of MCUPs more quickly and precisely than previously in routine clinical settings and might be useful for surgeons to determine the appropriate surgical procedure for resectable cases.

## CONCLUSION

Our case suggests that patients with localized MCUP include a prognostically favorable subpopulation of MCUP that may receive a survival benefit from induction therapy and definitive surgery.

## ACKNOWLEDGEMENTS

We thank Dr. Ryu Jokoji, Department of Diagnostic Pathology, Osaka Keisatsu Hospital, for assisting the diagnosis. We also thank Dr. Angela Morben, DVM, ELS, from Edanz (https://jp.edanz.com/ac) for editing a draft of this manuscript.

## DECLARATIONS

### Funding

None.

### Authors’ contributions

YS is the corresponding author and carried out the revision of the manuscript.

NT, YK, and NH carried out the review of medical records.

All authors have read and approved the final version of the manuscript being submitted.

The authors declare that the content of the manuscript has not previously been published elsewhere.

### Availability of data and materials

The authors ensure that all the required datasets are presented in the main manuscript with no additional supporting files.

### Ethics approval and consent to participate

The institutional review board of Osaka Keisatsu Hospital approved this case report (No. 1944).

### Consent for publication

Written informed consent was obtained from the patient for publication of this case report and any accompanying images.

### Competing interests

The authors declare that they have no competing interest.
